# Reference values for toe grip strength among Japanese adults aged 20 to 79 years: a cross-sectional study

**DOI:** 10.1186/1757-1146-7-28

**Published:** 2014-05-13

**Authors:** Daisuke Uritani, Takahiko Fukumoto, Daisuke Matsumoto, Masayuki Shima

**Affiliations:** 1 Department of Physical Therapy, Faculty of Health Science, Kio University, 4-2-2 Umaminaka, Koryocho, Kitakatsuragigun, Nara 6350832, Japan; 2 Department of Public Health, Graduate School of Hyogo College of Medicine, 1-1 Mukogawacho, Nishinomiya, Hyogo 6638501, Japan

**Keywords:** Aging, Muscle degeneration, Foot, Movement, Physical function, Sex, Flexor strength

## Abstract

**Background:**

No standardised method has been adopted for measuring toe-grip strength (TGS), and no reference values have been established for evaluating it. The present study investigated age-related changes in TGS and the association of TGS with various descriptive characteristics.

**Methods:**

TGS was measured in both feet of 1842 community-dwelling individuals aged 20–79 years using a toe-grip dynamometer. The participants were classified by decade into six age groups: 20–29, 30–39, 40–49, 50–59, 60–69, and 70–79 years. Correlations for TGS between the dominant and non-dominant sides were analysed according to decade and sex using Pearson’s correlation coefficient. The mean TGS and TGS-to-weight ratio (TGS/Wt%) were compared between sexes by each decade and among all decades by sex using two-way analysis of variance with post-hoc tests. To assess relationships between mean TGS and various descriptive characteristics, we determined Pearson’s correlation coefficient by sex and performed a stepwise multiple-regression analysis. Significance was set at 5%.

**Results:**

Correlations for TGS between the dominant and non-dominant sides were significant in all decades by sex, ranging from 0.73 for men in their 70s to 0.91 for women in their 50s. Mean TGS and TGS/Wt% significantly differed between the sexes in all decades and in all decades except the 40s, respectively. In men, the mean TGS and TGS/Wt% significantly decreased with aging after the 50s and 60s, respectively. In women, both the mean TGS and TGS/Wt% significantly decreased between the 40s and 50s and between the 60s and 70s. TGS significantly correlated with age, height, and weight in both sexes. The stepwise multiple-regression analysis revealed TGS was significantly associated with sex, age, height, and weight (adjusted R^2^ = 0.31).

**Conclusions:**

TGS was closely correlated between the dominant and non-dominant sides. TGS and TGS/Wt were significantly reduced with aging after the 50s in men and significantly reduced between the 40s and 50s and between the 60s and 70s in women. Age, sex, height, and weight accounted for only 30.8% of the variance in TGS. Therefore, other factors (e.g. toe flexibility, structural characteristics) should be considered for improving the accuracy of predicting TGS.

## Background

A sense of equilibrium and vision plays an important role in the control of posture and movement [1-3]. In addition, as the feet and toes are the only parts of the body connected to the ground, providing both tactile and pressure information through the plantar afferents [4], somatic sensation through these organs is also very important for various movements, including standing [5,6] and walking [4,7,8]. In particular, the toes dynamically control posture and movement as a result of their more rapid mobility compared to the feet, and can both generate propulsion force during walking and prevent forward falls [5,7,8].

Toe function is often represented as toe flexor strength in various studies. Some investigators have reported that toe flexor strength decreases with aging [5,9,10] and have found that low toe flexor strength is an important risk factor for falls among elderly individuals [11-13]. In addition, inadequate toe strength can result in hallux valgus or lesser toe deformities [13-15] that can also reduce balance [11] and increase the risk of falls among elderly persons [8,11]. Hence, valuating and strengthening the toe flexors is important for predicting and preventing toe deformities and physical dysfunction, particularly among the elderly.

To date, no standard has been established for evaluating toe flexor strength, in contrast to the determination of hand grip strength. Some investigators have defined toe flexor strength as pressure force on the ground as measured using force plates or a force platform [5,9,13]. However, these methods are potentially inconvenient as a result of the setup, portability, and costs in both the clinical and research settings. The towel-gathering exercise is currently prescribed in clinical practice as a strengthening exercise for the toe flexor muscles [16]. Within the context of this clinical exercise, changes in toe grip strength (TGS) could be assessed as an outcome of strengthening the toe flexor muscles.

We investigated the intra- and inter-rater reliability of toe-grip dynamometry for standardising TGS measurements [17]. One study found that the intra- and inter-rater reliability of measuring TGS using toe-grip dynamometry showed substantial to strong agreement [17]. However, age-related reference values for TGS have not been established, and the relation of TGS to various characteristics remains unclear. Therefore, we assessed age-related changes in TGS and the association of TGS with different descriptive characteristics.

## Methods

### Participants

Participants were 1842 community-dwelling individuals aged from 20–79 years [male, n = 618; female, n = 1224; mean age (standard deviation) = 56.6 (14.3) years]. Data were collected from April 2011 to April 2013. All of the participants were volunteers recruited from participants of municipal events conducted for the measurement of physical fitness and managed by Kashihara city and Koryo town, Nara, Japan. None of the participants had known neuromuscular or musculoskeletal pathologies, used walking aids, or had severe hallux valgus. Participants were screened prior to the measurements for the study protocol, and those using a walking device were excluded. Toe deformity was confirmed by inspection. Severe hallux valgus was defined as grade 4 according to the grading system by Garrow et al. [18]. At the municipal events where we collected data, the individuals were often older and female. The interest of these particular individuals in their own health or the concern of younger individuals about their jobs the following day might have influenced the decision to participate in the municipal events, and lead to older individuals and females representing a greater proportion of the study population than the younger individuals and males.

The Research Ethics Committee of Kio University (H23-8) approved the study, and all subjects provided prior written informed consent to participate in the study.

### Experimental protocol

**Figure 1:**
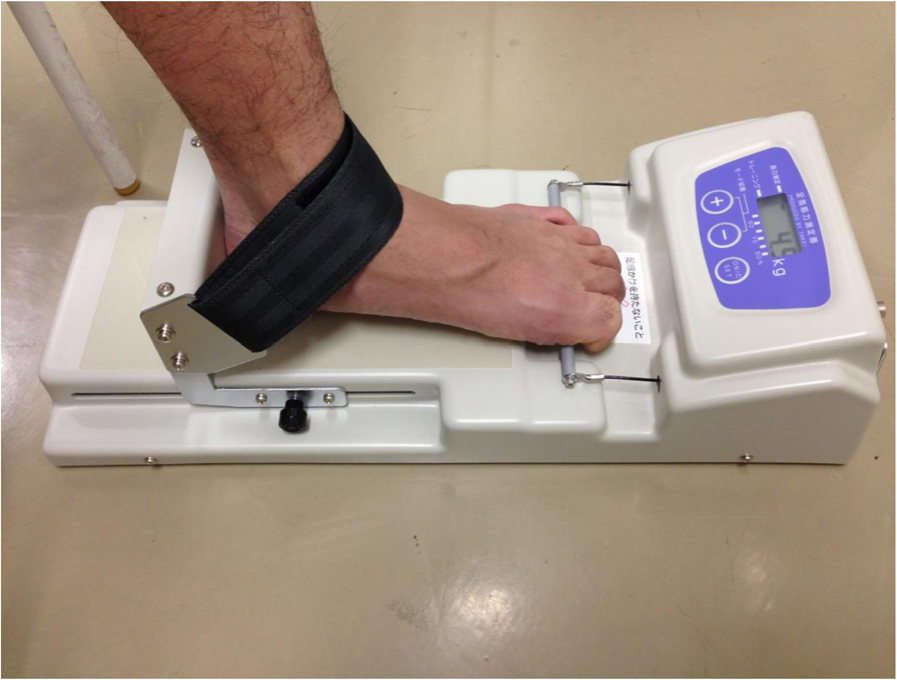
**Test of toe grip strength.** The first proximal phalanx is positioned at the grip bar, and the movable heel stopper is adjusted to fit the heel.

We measured TGS using a T.K.K.3362 toe-grip dynamometer (Takei Scientific Instruments, Niigata, Japan). The reliability of this device for individuals aged 20–79 years has been described [17]. The participants sat upright on a chair without leaning on the backrest throughout the TGS measurement. Both of the hips and knees were flexed about 90°, and the ankles were placed in the neutral position and fixed with a strap. The first proximal phalanx was positioned at the grip bar, and the heel stopper was adjusted to fit the heel of each participant. Some participants were not able to grip the bar with their fifth toe. However, since the muscle strength of the first toe has been reported to have the strongest association with TGS among toes [19], the first toe was used as a benchmark to set up the testing position. The bar was then gripped with maximal effort using the toes at maximal force for about 3 seconds (Figure 1). Testers stabilised the toe-grip dynamometer during the measurements. Participants practiced the test at submaximal effort before actual measurements were taken. The dominant foot was identified as that preferred for kicking a ball. Two TGS measurements were recorded for each set of toes in an alternating fashion. The set of toes to be measured first was randomly selected, and the TGS was subsequently measured for the other set. The third and fourth measurements (the second measurement for each set of toes) were conducted according to the same procedure. After the maximum strength from the two measurements for both sets of toes were recorded, the mean maximum strength for both sets (mean TGS) and the mean TGS-to-weight ratio (TGS/Wt%) were calculated.

### Statistical analysis

We analysed age-related changes in TGS by classifying the participants into six age groups according to decade: 20–29, 30–39, 40–49, 50–59, 60–69, and 70–79 years (Table 1). The correlations for mean TGS between the dominant and non-dominant sides according to decade and sex were calculated using Pearson’s correlation coefficient. Differences in mean TGS were determined using a two-way analysis of variance (sex × decade) followed by Tukey’s post hoc test. To assess relationships between mean TGS and various descriptive characteristics, we calculated Pearson’s correlation coefficient by sex and conducted a stepwise multiple regression analysis that included age, sex, height, and weight as explanatory valuables. The significance level was set at 5%.

**Table 1:** Characteristics of participants by decade and sex

**Decade/Sex**	**n**	**Age (y)**	**Height (cm)**	**Weight (kg)**
20s				
Man	69	23.9 (3.0)	171.0 (5.8)	65.1 (8.6)
Woman	57	23.5 (2.8)	159.1 (4.7)	52.9 (8.0)
30s				
Man	72	35.0 (2.9)	171.4 (6.0)	67.5 (9.8)
Woman	62	36.0 (2.6)	158.7 (6.2)	52.6 (7.7)
40s				
Man	101	44.1 (2.8)	170.5 (4.8)	68.3 (9.4)
Woman	155	44.9 (2.9)	157.8 (5.4)	53.6 (8.0)
50s				
Man	83	54.4 (3.1)	168.6 (5.1)	65.8 (9.8)
Woman	234	54.7 (2.9)	155.9 (5.4)	52.3 (8.9)
60s				
Man	177	64.7 (2.8)	166.0 (5.9)	63.3 (9.4)
Woman	493	64.4 (2.8)	152.7 (5.1)	51.7 (7.5)
70s				
Man	116	72.4 (2.3)	164.9 (4.9)	63.2 (7.8)
Woman	223	72.6 (2.4)	150.5 (5.4)	50.6 (7.6)

Age, height and weight are expressed as means (SD).

## Results

The descriptive characteristics of the participants according to decade and sex are shown in Table 1. Large differences were observed in the number of participants in each age group (e.g. 126 in the group aged 20–29 years and 670 in the group aged 60–69). Similarly, large differences in the number of males and females were seen in some of the age brackets (Table 1).

In both sexes, TGS was significantly correlated between the dominant and non-dominant sides in all decades (p < 0.01) and ranged from 0.73 in men in their 70s to 0.91 in women in their 50s (Table 2).

**Table 2:** **Correlation coefficients for TGS between dominant and non**-**dominant side**

	**Total**	**20s**	**30s**	**40s**	**50s**	**60s**	**70s**
Men	0.89	0.90	0.85	0.90	0.87	0.85	0.73
Women	0.90	0.86	0.88	0.89	0.91	0.90	0.90

*TGS*, toe grip strength. All correlations significant at p < 0.01.

The sex-decade interaction was significant (F_5, 1830_ = 5.963, p < 0.01), and the main effects were sex (F_1, 1830_ = 359.988, p < 0.01) and decade (F_5, 1830_ = 59.514, p < 0.01). Differences in the mean TGS or TGS/Wt% between the sexes were significant in all decades (p < 0.01) and in all decades except the 40s (20s, 50s, and 70s, p < 0.01; 30s and 60s, p < 0.05), respectively (Table 3).

**Table 3:** **Mean toe grip strength and mean toe grip strength**/**weight**

	**Mean TGS (kg)**		**Mean TGS/Wt (%)**	
	**Men**	**Women**	**Men**	**Women**
20s	16.9 (6.0)^†^	10.4 (4.1)	26.5(10.2)^†^	20.0 (7.9)
30s	17.1 (5.0)^†^	11.5 (4.9)	25.7 (7.8)^*^	22.1 (9.4)
40s	15.8 (6.2) ^†^	11.6 (4.5)	23.4 (9.0)	21.8 (8.0)
50s	14.4 (4.8)^†b^	8.9 (3.6)^BC^	22.2 (7.8)^†^	17.3 (6.9)^BC^
60s	12.0 (4.5)^†ABCD^	8.9 (4.0)^BC^	19.3 (7.5)^*ABC^	17.5 (8.2)^BC^
70s	10.4 (3.3)^†ABCDE^	7.3 (3.6)^ABCDE^	16.5 (5.1)^†ABCDE^	14.6 (7.4)^ABCDE^

Data expressed as means (standard deviation). TGS, toe grip strength; Wt, Weight.

*p < 0.05 and ^†^p < 0.01 vs. women. p < 0.01: A, B, C, D, E vs. 20s, 30s, 40s, 50s and 60s, respectively; ^b^p < 0.05 vs. 30s.

The mean TGS was significantly lower for men in their 50s than men in their 30s (p < 0.05). Mean TGS was significantly lower for men in their 60s than men in their 20s to 50s (all p < 0.01). The mean TGS was significantly lower for men in their 70s than those in their 20s to 60s (all p < 0.01). The mean TGS/Wt% was significantly lower for those in their 60s than those in their 20s to 40s (all p < 0.01), and for those in their 70s than for those in their 20s to 60s (all p < 0.01). Mean TGS was significantly lower for women in their 50s and 60s than women in their 30s and 40s (all p < 0.01), and for women in their 70s than those women in their 20s to 60s (all p < 0.01). The trends of mean TGS/Wt% were similar to those of TGS.

The mean TGS significantly correlated with age (men, r = −0.43; women, r = −0.28), height (men, r = 0.33; women, r = 0.31), and weight (men, r = 0.19; women, r = 0.16) (all p < 0.01; Table 4). The stepwise multiple regression analysis revealed that age (β = −0.24), sex (male: 0, female: 1, β = −0.17), height (β = 0.23), and weight (β = 0.08) were associated with TGS (all p < 0.01; adjusted R^2^ = 0.31; Table 5).

**Table 4:** **Correlation coefficients between mean TGS and age**, **height and weight**

		**Age**	**Height**	**Weight**
TGS	Men	−0.43	0.33	0.19
	Women	−0.28	0.31	0.16

*TGS*, toe grip strength; all correlations significant at p < 0.01.

**Table 5:** Multiple regression analysis of toe grip strength

	**Regression coefficient**	**Standard deviation**	**β**	**p**
Intercept	−6.26	3.40		0.07
Age (y)	−0.09	0.01	−0.24	p < 0.01
Sex (M,0; F,1)	−1.86	0.33	−0.17	p < 0.01
Height (cm)	0.13	0.02	0.23	p < 0.01
Weight (kg)	0.04	0.01	0.08	p < 0.01

R = 0.56, adjusted R^2^ = 0.31.

## Discussion

We identified strong correlations in TGS between the left and right sides of the body across all decades in both sexes (from 0.73 in men in their 70s to 0.91 in women in their 50s). Therefore, in the remainder of the study analysis, we used the mean TGS of both sides for presenting the values of TGS.

Chhibber and Singh [20] found significant asymmetry in the muscle weight of the lower limbs based on a cadaver study and postulated that the functional dominance of one limb over the other had resulted in the asymmetry. Riskowski et al. [21] found that asymmetric foot function and the degree of asymmetry became reduced with advancing age. Based on our findings, there were strong correlations in TGS between the different sides of the body for both sexes, although an increasing asymmetry was observed with age for men but not for women.

The mean TGS was significantly weaker in women than men across all decades, a finding that is similar to those for hand grip strength [22], knee extension, and flexion torque [23]. The mean TGS/Wt% was also decreased in women compared to that in men, except in the 40s. This result indicates that weight might not influence the gender differences in TGS.

The mean TGS decreased with aging in both men and in women. The mean TGS in men started to decrease significantly in the 50s and then significantly decreased at ≥60 years of age, whereas the decrease started between the 40s and 50s in women. The mean TGS in both men and women in their 70s was significantly weaker than in those in their 20s to 60s. Endo et al. [5] found that the maximum toe flexor muscle strength was significantly different between younger and older individuals. Additional studies have also reported that aging is associated with reduced toe plantarflexion strength [9,10]. The results of the present study are consistent with these findings. Previous studies have also described a decline in muscle strength and muscle mass with aging [24,25]. Wilmore [26] found that muscle mass and strength declines relatively slowly from 20 to 50 years of age, but this decrease is most apparent between the ages of 50 and 60 years.

Previous studies have described the cellular and molecular mechanisms of age-related muscle weakness [27] and have characterised the changes in muscle fibre composition during aging [28]. In a study of 15–83-year-old men, Lexell et al. [29] observed the average number of fibres in the vastus lateralis muscle showed no changes between 18 and 50 years of age. However, the mean number of fibres in those who had reached 80 had decreased to 50% compared to that in younger men. Campbell et al. [30] similarly found that the number of motor units remained constant from 5 to 50 years of age but subsequently decreased in a linear fashion with a zero intercept at 95 years of age.

Decreasing muscle strength may differ between men and women. A relatively slow decline in muscle mass and strength occurs between 20 to 50 years of age, becoming apparent between 50 and 60 years [26]. This decline has been reported to be greater in women than in men [24]. Samson et al. [31] reported knee extensor and handgrip strength gradually decreases between 20 and 80 years of age in men but steeply declines in women after the age of 55 years. This decline in strength was accelerated in women around menopause. Muscle weakness increases with age and tends to become more pronounced in women when oestrogen and progesterone production declines at menopause [32]. Therefore, menopause may also influence the decrease in TGS among women.

In the present study, TGS significantly decreased in both sexes in the 70s. Frontera et al. [33] found significantly lower amounts of fat-free mass and muscle mass among 65–78 year olds than among 45–54 year olds and 55–64 year olds. Meanwhile isokinetic muscle strength of the knee and elbow extensor and flexor were significantly lower in 65–78-year-old men and women than in 45–54-year-old men and women. Hence, our results are consistent with these previous findings. The results of the multiple regression analysis also suggested that TGS is influenced more by age than body composition.

In the present study, both mean TGS and mean TGS/Wt% decreased with aging. Frontera et al. [33] reported that age-related differences in the isokinetic strength of the elbow and knee extensors and flexors were not significant in any muscle group, with the exception of knee extensors tested at 240°/sec when strength was adjusted for fat-free mass or muscle mass. The present results are not consistent with these findings. Menz et al. [9] found that normalised toe plantarflexion strength differed between younger and older individuals. In the present study, the body weight did not significantly change with aging in men or women. However, the mean TGS/Wt% was observed to decrease with aging. This suggests that decreasing TGS influenced the age-related change in TGS/Wt%. Fat-free mass or muscle mass might have decreased, while body fat might have increased in our participants, which would have resulted in the absence of a significant change in body weight and a decrease in TGS with age. Endo et al. [5] reported that when normalised by body size (weight × height), the gender difference in maximum toe flexor muscle strength no longer achieved statistical significance. However, the definition of body size differed between the current study and this previous one.

In addition to determining reference TGS values by decade, we investigated the association of TGS with various descriptive characteristics. Bohannon [34] reported reference values with high coefficients of determination for extremity muscle strength, as determined by a hand-held dynamometry, among adults aged 20 to 79 years based on sex, age, and weight. Meanwhile, according to a stepwise multiple logistic regression analysis in the present study, age, sex, height, and weight were independently associated with TGS, with an adjusted coefficient of determination of 0.31 for TGS. That is, 31% of the variance was accounted for based on the sex, age, height, and weight. Thus, other factors besides the evaluated descriptive characteristics might have affected TGS. Toe deformities are frequent even among able-bodied populations. Mickle et al. [13] reported that individuals with hallux valgus and lesser toe deformities had weaker flexor muscles of the associated toes. Although none of our participants were unable to grip with their toes as a result of severe deformities, slight deformities might have influenced TGS. Furthermore, lack of toe motion and change of foot structure might be influenced by age as compared with the fingers and hands. The lack of a range of motion is often unrecognised in the toes, unlike the fingers, and might have also influenced TGS. Scott et al. [10] reported older people have flatter/more pronated feet and found a reduced range of motion in the first metatarsophalangeal joint. Therefore, the effects of toe and foot structures require more detailed investigation. In addition, physical activity and preferences for types of sports and jobs might also influence TGS as well as other types of muscle strength [35-37]. However, we did not investigate these factors in the present study. Therefore, multiple factors affecting TGS will require consideration in future studies.

This study has several limitations. First, some participants were not able to grip the bar with their fifth toe owing to differences in toe length. Therefore, in some participants, TGS was not presented as the strength of all the toes. However, as the muscle strength of the first toe has been reported to show the strongest association with TGS among toes [19], the first toe was used as a benchmark to set up the testing position. Second, the method used for measuring TGS in this study could not control for the grip force produced by the midfoot, and future studies should control for arch contraction and its contribution to TGS. Third, participants were unable to be assigned randomly since this study was performed based on events conducted by a municipal government. Hence, participant characteristics might have been biased in terms of interest in their health. This could be one of the reasons for the uneven participant distribution among the decades. Moreover, the small sample size of the younger and male participants must be taken into account when the results of our study are applied in clinical practice. Fourth, our data were obtained from Japanese people reducing the generalizability of these TGS measures to other ethnic groups. We investigated TGS among able-bodied community-dwelling individuals aged 20 to 79 years. Future studies should assess individuals aged <20 and >80 years as well as persons with disabilities. Finally, although we assessed the association of TGS with sex, age, height, and weight, the coefficient of determination was insufficient. Future studies must take into account other relative factors to clarify contributing factors of TGS with higher accuracy.

## Conclusions

This study was the first to investigate reference values for TGS by sex and decade using a toe-grip dynamometer, which has broad utility. The results provided reference values for TGS in community-dwelling Japanese men and women in their 20s to 70s. We identified sex difference in TGS in all decades and a decrease in TGS with age after the 50s in both sexes. We also clarified an association of TGS with age, sex, height, and weight. These results may contribute to future comparative studies evaluating toe function. However, as TGS might be influenced by many other factors, such as arch height and foot and toe deformity, these should be taken into account to predict TGS more accurately.

## Abbreviations

TGS: Toe grip strength; TGS/Wt%: TGS-to-weight ratio.

## Competing interests

The authors declare that they have no competing interests.

## Authors’ contributions

DU participated in the study design; data acquisition, analysis, and interpretation; and drafting the manuscript. TF participated in the study design, data acquisition, and helped to draft the manuscript. DM participated in data acquisition and helped to draft the manuscript. MS helped with the statistical analysis, data interpretation, and drafting the manuscript. All authors read and approved the final version.
